# Dynamic remodeling of centrioles and the microtubule cytoskeleton in the lifecycle of chytrid fungi

**DOI:** 10.1091/mbc.E24-12-0577

**Published:** 2025-08-20

**Authors:** Alexandra F. Long, Krishnakumar Vasudevan, Andrew J.M. Swafford, Claire M. Venard, Jason E. Stajich, Lillian K. Fritz-Laylin, Jessica L. Feldman, Tim Stearns

**Affiliations:** a Department of Biology, Stanford University, Stanford, CA 94305; b Department of Biology, University of Kentucky, Lexington, KY 40508; c Department of Biology, Middlebury College, Middlebury, VT 05753; d Department of Microbiology and Plant Pathology, University of California Riverside, Riverside, CA 92521; e Department of Biology, University of Massachusetts Amherst, Amherst, MA 01003; f Laboratory of Cellular Dynamics, Rockefeller University, New York, NY 10065

## Abstract

Cell movement and division are complex behaviors driven by a dynamic internal cytoskeleton. The molecular components and principles of cytoskeletal assembly are well studied, but less is known about cytoskeletal remodeling events, including how centrioles transition from ciliary base to centrosome. Here, we address this using the chytrid *Rhizoclosmatium globosum*, a zoosporic fungus that has centrioles and cilia, lost in most fungal lineages. Chytrids undergo reorganization of their microtubule cytoskeleton as they grow from zoospore to multinucleated coenocyte. We use evolutionary comparison, RNA-sequencing, and expansion microscopy to understand this reorganization and further develop this organism as a model for evolutionary cell biology. We find that when motile zoospores transition to sessile sporangia, cilia are retracted into the cytoplasm and degraded, while centrioles detach from the ciliary axoneme yet persist. During the mitotic cycles, short centrioles are associated with a centrosome-like microtubule-organizing center (MTOC) and a dense microtubule array at the spindle pole. After the mitotic cycles, centrioles elongate and form cilia, driven by transcription of genes associated with centriole maturation and ciliogenesis, and microtubule bundles are reorganized. Thus, in chytrids structural remodeling of the centriole is temporally coupled to specific changes in cytoskeletal organization over the coenocytic lifecycle.

## INTRODUCTION

Cells organize and rearrange their contents in space and time to perform complex behaviors such as dividing and swimming, driven by their dynamic cytoskeleton. The microtubule cytoskeleton is patterned by microtubule-organizing centers (MTOC). Defects in the spatial and temporal patterning of the microtubule cytoskeleton can compromise cell division, motility, and signaling, causing diseases from ciliopathies to cancer (Reiter and Leroux, 2017).

One important and highly conserved microtubule structure in the eukaryotic cell is the cilium/flagellum, a long hair-like protrusion that has roles in signaling and motility. The base of the cilium is a barrel-shaped centriole made from microtubules, from which emerges the ciliary axoneme, a long and highly patterned bundle of specialized microtubules. Organisms or cell types with cilia often alternate states for the centriole between serving as the base of the cilium and as the center of the centrosome. Centrosomes are MTOCs that have a pair of centrioles surrounded by a matrix of pericentriolar material (PCM) that nucleates and organizes arrays of microtubules in interphase and the spindle during mitosis. For centrioles to play these dual roles as part of cilia and centrosomes, they must be uncoupled from their accompanying structures and remodeled over time (Kalbfuss and Gönczy, 2023). This problem is common to all cells that transition from a ciliated to a nonciliated state, from unicellular organisms to animal ciliated cells that participate in the cell cycle. Although decades of work have focused on the components and rules for building cytoskeletal structures, we know comparatively less about the molecular and structural basis for how MTOCs are remodeled to enable distinct cell states and functions. In part this is because much of what we know about MTOC remodeling comes from the study of animal centrosomes in cultured cycling cells. To understand more about how cells remodel MTOCs in vivo, we need to study organisms and cell types that readily undergo cilium and centrosome remodeling events during their cell cycle or lifecycle.

Many unicellular organisms across the eukaryotic tree exhibit particularly striking examples of remodeling during complex developmental transitions. We have chosen to study these questions in chytrid fungi (Powell, 1980; 2017), a group of fungi that have motile cilia and centrioles common to other eukaryotic lineages. Chytrids (phylum: Chytridiomycota) are a member of the zoosporic fungi, which retain these complex microtubule structures lost in the rest of the fungal lineages. Thus chytrids occupy a key phylogenetic position to shed light onto MTOC biology and evolution (Yubuki and Leander, 2013; Ito and Bettencourt-Dias, 2018). Chytrids extensively remodel their microtubule cytoskeleton over their lifecycle as they develop from a motile zoospore to a stationary multinucleated sporangium. They retract the long cilium of the zoospore into the cell body, then degrade this massive structure (Koch, 1968; Venard et al., 2020; Laundon et al., 2022) and remodel the centrioles to form a mitotic MTOC, going through multiple mitoses without cell division to create a large coenocytic sporangium. In this common cytoplasm, one new cilium is assembled per nucleus, followed by subdivision into new zoospores that are released to the environment.

Historically, chytrid cell biology has focused extensively on taxonomy, and only recently have there been efforts to map molecular changes over the lifecycle (Rosenblum et al., 2008). Chytrids play critical ecological roles in aquatic ecosystems (Gleason et al., 2017) with many living as parasites on organisms from amphibians to algae (Longcore et al., 1999) using cellular mechanisms involving remodeling of the chytrid cytoskeleton (Brody et al., 2025). Tools for genetic transformation are beginning to be developed for a few chytrid species (Medina et al., 2020; Swafford et al., 2020; Kalinka et al., 2024). It has been challenging to visualize chytrid cells using immunofluorescence due to the small size of zoospores and the complex fungal cell wall of the sporangium. Thus, we currently lack molecular and structural understanding of how chytrids execute these dramatic cytoskeletal changes in normal lifecycles and in pathogenic contexts, and, specifically, how the cilium–centriole complex is disassembled during ciliary retraction and reformed during ciliogenesis.

To address these questions, we use the nonpathogenic chytrid fungus *Rhizoclosmatium globosum* (*Rg*) together with RNA-sequencing and high-resolution ultrastructure expansion microscopy (U-ExM) to characterize microtubule reorganization during the coenocytic lifecycle. Many chytrid species retain a complex actin cytoskeleton that enables amoeboid crawling in addition to swimming with a motile cilium (Prostak et al., 2021). *Rg* has a reduced actin cytoskeleton and does not exhibit amoeboid motility, has a highly synchronous 20 h lifecycle, a sequenced genome, and exhibits representative chytrid cell architecture and development making it an excellent model for specifically studying remodeling of the microtubule cytoskeleton (Berger et al., 2005; Mondo et al., 2017; Powell, 2017; Laundon and Cunliffe, 2021; Laundon et al., 2022). Here, we find that *Rg* cells have complex MTOCs at different lifecycle stages and dynamically remodel centrioles and MTOCs as they transit between ciliogenesis, interphase, and mitosis.

## RESULTS AND DISCUSSION

### Developing tools to study chytrid fungi

**FIGURE 1:**
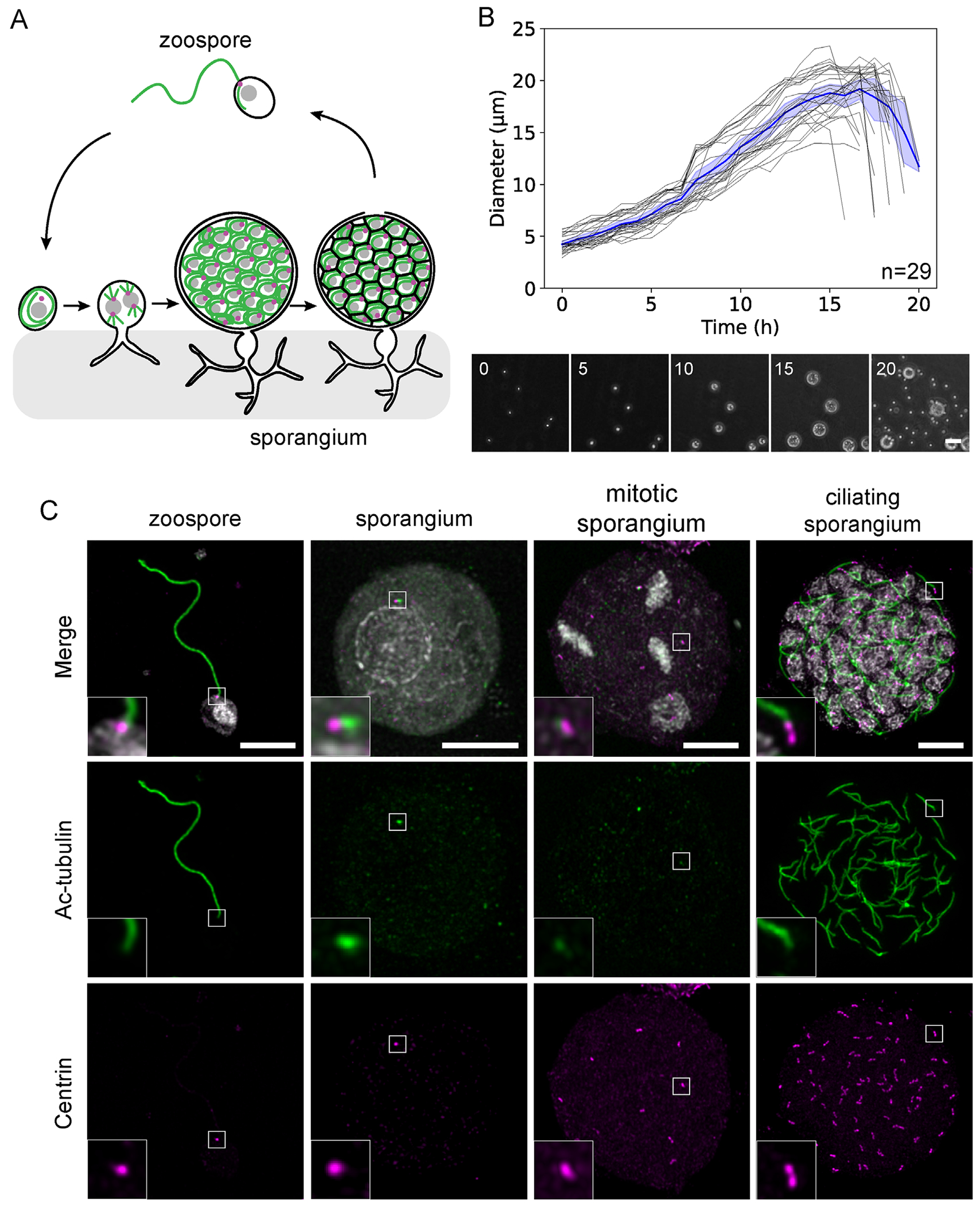
*R. globosum* has a synchronous coenocytic lifecycle. (A) Cartoon of the lifecycle of a chytrid fungus beginning from a motile zoospore (top) that retracts its cilium (green) into the cell body and detaches centrioles (pink) before progressing through mitotic cycles without cytokinesis. Rhizoids at the base of the sporangium attach to the substrate. Ultimately the coenocytic sporangium contains dozens of nuclei and centrioles that undergo ciliogenesis in the shared cytoplasm before cellularizing into individual zoospores. (B) Time course of *R. globosum* lifecycle imaged with phase-contrast microscopy from zoospore plating to new zoospore release; scale bar, 100 μm. Quantification of raw (black) and average (blue) sporangium diameter over the lifecycle showing synchronous development (*n* = 29 sporangia from three independent experiments). (C) Immunofluorescence characterization of subcellular structures over the chytrid lifecycle (DAPI; white, acetylated-tubulin; green, centrin; magenta). The zoospore has a cilium and centriole, while the sporangium has degraded the ciliary axoneme but not the centriole and increases in size as it matures and enters mitosis. This example shows synchronous metaphase spindles of the second mitosis. Synchronous ciliogenesis occurs after the end of the mitotic cycles; scale bar, 10 μm (insets, 3.5x enlarged).

To begin to develop chytrid fungi as a system for studying centriole and MTOC remodeling, we first characterized the lifecycle of *Rg* strain JEL800 using light microscopy, from initial zoospore formation to the release of new zoospores. A schematic (Figure 1A) illustrates the lifecycle, starting from the motile zoospore, which undergoes cilium retraction and centriole detachment, followed by mitotic divisions without cytokinesis, forming a multinucleate sporangium. Hyphal-like rhizoids anchor the sporangium to the substrate as it enlarges and develops. Eventually after building new cilia from each pair of centrioles, the sporangium cellularizes into separate zoospores that are then released (Medina et al., 2025). Phase-contrast microscopy (Figure 1B) confirmed the temporal dynamics and synchrony of this process in this strain.

Immunofluorescence is an essential tool for studying the cytoskeleton, but it has been technically challenging in chytrids due to the chitin-based cell wall present during much of the lifecycle. Thus, we developed an optimized protocol for immunofluorescence using chitinase treatment and physical freeze-cracking of *Rg* sporangia. This protocol enables labeling of the *Rg* microtubule cytoskeleton at all lifecycle stages using immunofluorescence (Figure 1C). We characterized markers for centrioles and cilia in chytrid zoospores and sporangia. First, we examined a cilium and centriole marker using an antibody against acetylated-tubulin, a posttranslationally modified form of tubulin, that decorates the stable microtubule population (Piperno et al., 1987). As is seen in other systems, in *Rg* axonemal microtubules are highly acetylated (Figure 1C) and acetylation is greater on the centrioles at the poles of the mitotic spindle, relative to the microtubules of the spindle itself in mitotic sporangia. To specifically localize centrioles at each stage, we used an antibody against centrin, a conserved protein that plays a role in centriole duplication and centriole tethering in diverse eukaryotes (Taillon et al., 1992; Levy et al., 1996; Zhang and He, 2012). *Rg* contains a predicted ortholog of centrin and anti-centrin antibody labeled (Figure 1C) centrioles in both zoospores and sporangia. Thus chytrids have highly conserved components of the microtubule cytoskeleton and established molecular markers allow us to visualize the microtubules of the centriole and cilium throughout the chytrid lifecycle.

### Bioinformatic characterization of chytrid centriole-associated genes

**FIGURE 2:**
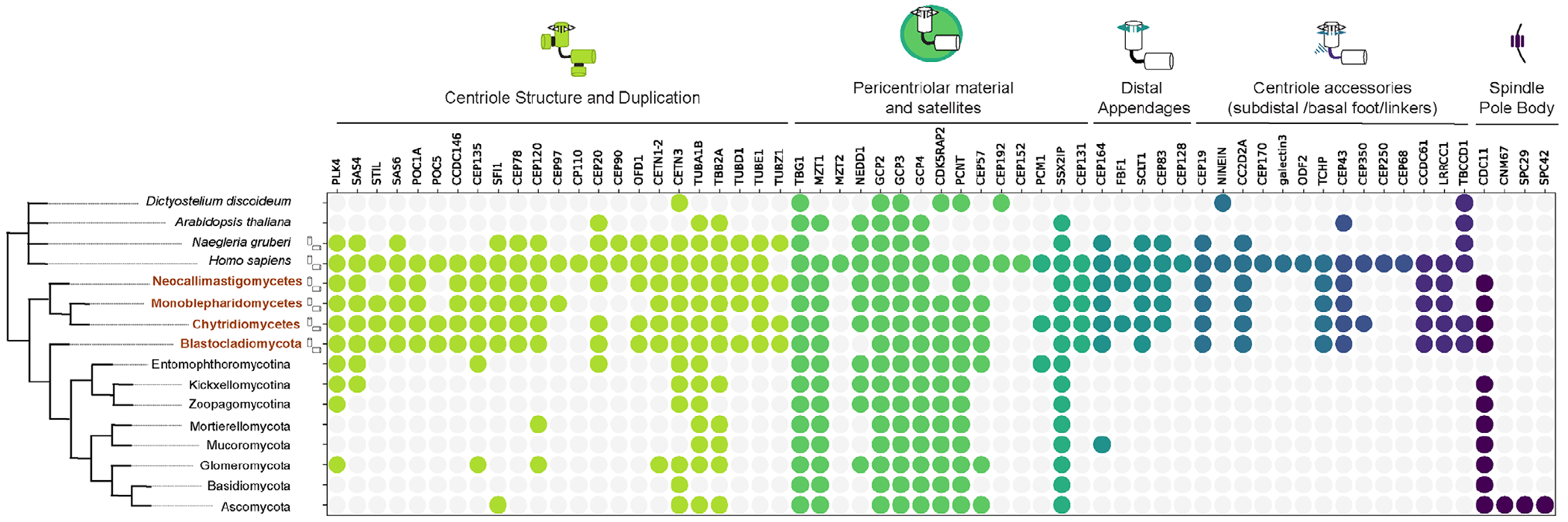
Broad conservation of centriole and centrosome-associated genes in zoosporic fungi. Orthologs of genes associated with different centriole and centrosome features across (left) four outgroups (gray, top) and fungal phyla and subphyla (bottom). Each colored circle denotes that there was at least one ortholog assignment in species of the associated fungal clade compared with four outgroups that include two species with (*H. sapiens, N. gruberi*) and two species without (*A. thaliana, D. discoideum*) centrioles and motile cilia. Organisms known to have centrioles and motile cilia are indicated with centriole cartoons and include 4 clades of zoosporic “chytrid” fungi (brown) that share a set of conserved centriole and cilium-associated genes across functional categories (light green: centriole structure and duplication; green: PCM and centriole satellites; teal: centriole distal appendages; blue/purple: centriole accessory structures, including subdistal appendage and basal feet as well as fibrous linkers).

The zoosporic fungi have retained centrioles and cilia (Hodges et al., 2010; Galindo et al., 2021), whereas other fungal lineages have replaced centrioles and cilia with a noncentrosomal MTOC called the spindle pole body, which has few components in common with animal centrosomes (Ito and Bettencourt-Dias, 2018). We took a comparative approach to determine which MTOC components are conserved in chytrids, using OrthoFinder (Emms and Kelly, 2015; 2019). We used a set of genes encoding proteins associated with mammalian centrioles and PCM as well as fungal spindle pole bodies across all fungal lineages with ciliated zoospores (Figure 2). We find that zoosporic fungi share many conserved genes with metazoans involved in core centriole structure as well as MTOC and basal body functionalization that are not present in other fungi, confirming their utility as a model for studying centriole and MTOC evolution.

Centrioles have a highly conserved, polarized structure that is closely linked with their function. At the base of the centriole are structures associated involved in tethering centrioles to other centrioles and organelles such as the nucleus. At the distal end of the centriole, from which the axoneme extends, are proteins that functionalize the structure to bind specific membranes and build the cilium. Chytrids have a large set of core structural components as well as proteins involved in centriole duplication and elongation (e.g., CPAP, CEP120). They also have genes for the distal appendage proteins that facilitate ciliogenesis and membrane docking, consistent with structures observed in transmission electron micrographs (TEM) that closely resemble the distal appendages of animal centrioles (Longcore et al., 1999; Longcore and Simmons, 2020). Chytrids have some orthologs of other distal centriole components (Kumar and Reiter, 2021) (e.g., CC2D2A, TCHP, CEP19, and FOP) and fibrous linkers (e.g., TBCCD1, LRRCC1/VFL1, CCDC61, and VFL3). Other electron-dense structures surrounding chytrid centrioles are similar within chytrid clades and have long been used for taxonomic classification but their function and molecular identity are not known as they do not closely resemble structures in animal cells such as the subdistal appendages of primary cilia or basal feet of motile cilia (Barr, 1981; Barr and Désaulniers, 1988; James et al., 2006; Kumar and Reiter, 2021).

With respect to components of microtubule structure and organization, chytrids have orthologs of the main tubulin family members, including alpha, beta, gamma-tubulin as well as epsilon and zeta-tubulin (Chang et al., 2003; James et al., 2006; Turk et al., 2015; Kumar and Reiter, 2021). Most other fungi lack the expanded tubulin family members, zeta, epsilon, and delta-tubulin, while this “ZED” module (Turk et al., 2015) is fully present in three of the four clades of zoosporic fungi, reduced in the Chytridiomycota, and absent in *Hyaloriphidium curvatum* that has lost cilia (Ustinova et al., 2000) (Supplemental Figure S1). Chytrids have a conserved suite of adaptors involved in microtubule nucleation and organization, including NEDD1, MZT1 and a larger family of gamma-tubulin complex proteins consistent with most other eukaryotes, suggesting that the core microtubule nucleation module is conserved. Genes for PCM proteins are in general less conserved across eukaryotes than those of the centriole and. Similar to yeasts (e.g., *Saccharomyces cerevisiae* and *Schizosaccharomyces pombe*), chytrids lack clear orthologs of most animal PCM components (e.g., pericentrin, CDK5RAP2, CEP57 but not CEP192 or CEP152), but do have proteins with short conserved domains found in these proteins, suggesting related function. Chytrids do not have proteins of the core modules of spindle pole bodies found in other fungi (e.g., SPC29, SPC42) (Hodges et al., 2010; Ito and Bettencourt-Dias, 2018; Galindo et al., 2021), consistent with the absence of these structures in chytrid cells.

In sum, we have determined the genomic signature of both chytrid centrioles and MTOCs. Chytrids have substantially more shared modules with metazoan centrioles than with fungal spindle pole bodies and retain many conserved genomic signatures of centrosomes such as modules involved in organizing and scaffolding PCM and nucleating microtubules.

### Transcriptional signature of centriole maturation and ciliogenesis

**FIGURE 3:**
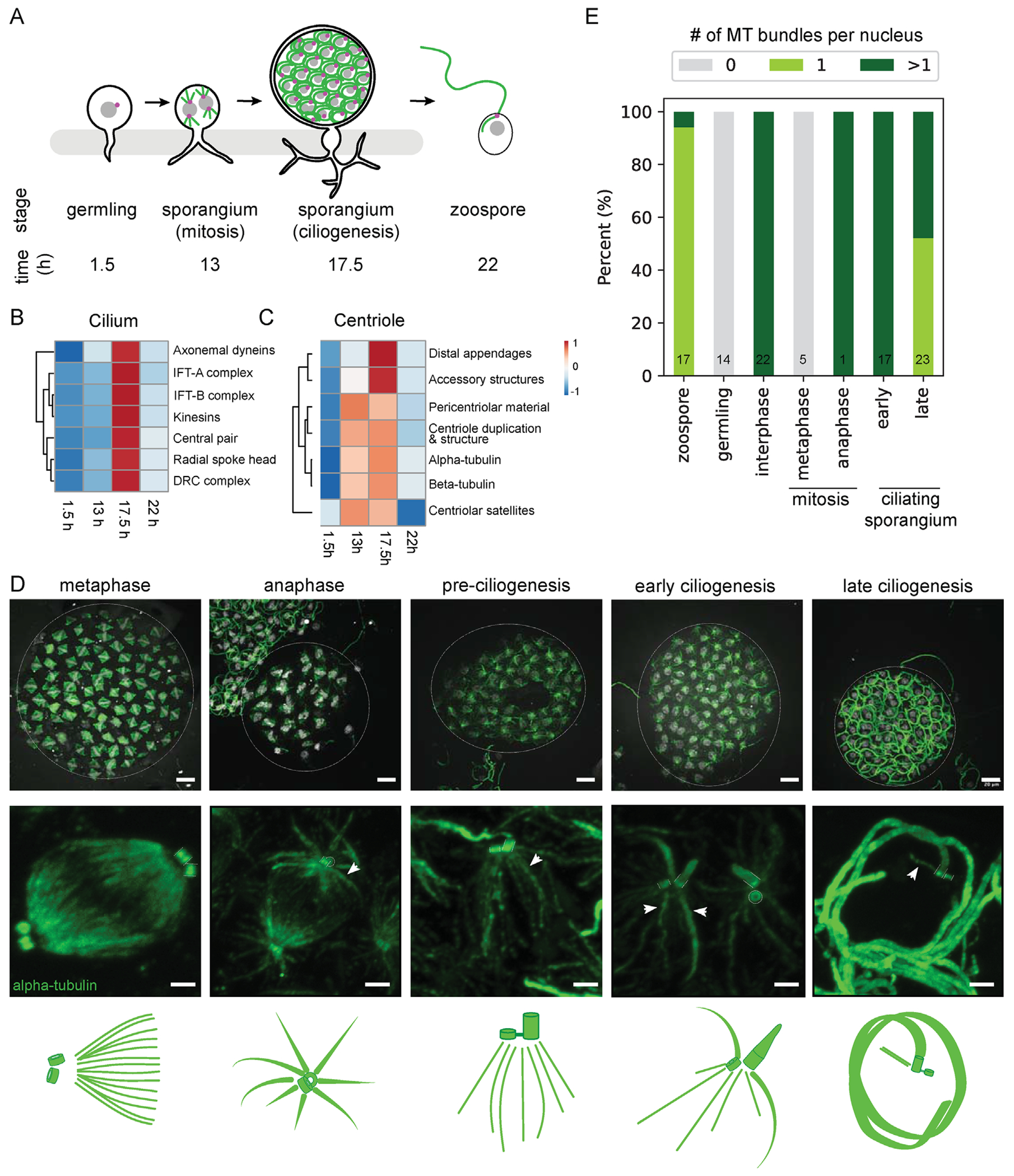
Centriole and MTOC architecture are remodeled during mitosis and ciliogenesis. (A) Timepoints in the chytrid lifecycle used for RNA-sequencing to probe key transitions (1.5 h early sporangium/germling, 13 h mitotic sporangium, 17.5 h ciliating sporangium, and 22 h new zoospores). (B and C) Heatmap of average RNA transcript abundance (FPKM, Fragments Per Kilobase of transcript per Million mapped reads) for groups of cilium and centriole-related genes at each timepoint. Cilium-related transcripts are upregulated at 17.5 h during ciliogenesis. Centriole related transcripts are upregulated from 13 to 17.5 h during mitosis and ciliogenesis, while centriole accessory structures associated with distal maturation are specifically upregulated at ciliogenesis. (D) Expansion microscopy characterization of chytrid MTOCs during mitosis and ciliogenesis (DAPI; white, alpha-tubulin; green). Scale bars 20 μm expanded (expansion factor ~4.5 fold) and 2 μm for insets. Insets and summary cartoons show a single nucleus and associated cytoskeleton. Centrioles are short and orthogonal during mitotic divisions and oriented end on to the spindle pole. One centriole elongates at the start of ciliogenesis. MTOC architecture changes over the lifecycle from no astral microtubules at metaphase, large microtubule arrays in anaphase and during ciliogenesis, and a single microtubule bundle before zoospore release. (E) Fraction of sporangia with each class of microtubule architecture at different lifecycle stages (*n* = number of sporangia from three to five independent experiments).

Given that zoosporic fungi retain many of the genetic modules associated with centrosomes, we sought to characterize the transcriptional profile of MTOC-related genes and the structure of MTOCs over the lifecycle, including mitosis and in the transition to ciliogenesis. We used RNA-sequencing to determine the transcriptional profiles associated with MTOC remodeling, using *Rg* cells at four timepoints after plating fresh zoospores: 1.5 h; germling, 13 h; mitotic sporangia, 17.5 h; ciliating sporangia, and 22 h; new zoospores (Figure 3A; Supplemental Figure S2A). We found a core set of transcripts at all lifecycle stages, as well as hundreds of transcripts unique to each of these four lifecycle stages (Supplemental Figure S1B). Overall we found that there is an ordered transcriptional program underlying the different lifecycle stages (Rosenblum et al., 2012; Laundon et al., 2022) (Supplemental Figure S2, C–F).

Ciliating sporangia have a strong transcriptional signature associated with ciliogenesis (Figure 3B), in which components of the axoneme and modules required for ciliary beating are upregulated. We also looked for the signature of centrioles and their accessory structures, informed by our bioinformatic mapping (Figures 2 and 3C). Transcripts for centriole-associated proteins are upregulated during the mitotic cycles and ciliogenesis. Specifically, distal appendage components and subdistal appendage/basal foot components are strongly upregulated during ciliogenesis compared with the mitosis phase, suggesting that, unlike in cycling mammalian cells, chytrid centrioles likely gain distal appendages just prior to ciliogenesis. This is consistent with the lack of distal appendage-like densities in TEM images of centrioles in mitotic chytrids (Longcore et al., 1999). We did not observe expression level differences in PCM components, consistent with posttranslational regulation of PCM expansion common in other systems (Breslow and Holland, 2019). Overall, we find ordered transcriptional programs associated with key lifecycle transitions into mitosis and ciliogenesis and specific modules associated with centriole remodeling and maturation.

### MTOC remodeling from mitosis to ciliogenesis

MTOCs have a variety of microtubule architectures across cell types or organisims. Centrioles at the base of cilia can also pattern cytoplasmic microtubules; this an occur via “rootlet” microtubule bundles that emanate from the side of specific microtubules in the centriole barrel (Azimzadeh, 2021) or as an aster of microtubules that radiates from the PCM. In chytrids, microtubules have been observed radiating from centrioles at the base of cilia (James et al., 2006) as well as from centrioles at the spindle pole during mitosis, but the morphology of the mitotic MTOC in chytrids and how this structure changes at the end of the mitotic cycles to reform cilia have not been systematically characterized.

To investigate the morphology of the mitotic MTOC in chytrids we had to overcome the challenges of the small size and low permeability of chytrid sporangia. We used ultrastructure expansion microscopy (U-ExM) (Gambarotto et al., 2019), a technique that physically enlarges a sample before antibody staining, yielding an increase in spatial resolution proportional to the expansion factor as well as improved permeabilization and lower background. This is the first use of expansion microscopy in zoosporic fungi and this technique is just beginning to be used in other fungal species (Götz et al., 2020; Hinterndorfer et al., 2022). By modifying the standard U-ExM protocol to be compatible with the thick chitinous cell wall of chytrids, we achieved an average expansion factor of 5.5 ± 0.2 (mean ± std) (Supplemental Figure S3, A and B).

Using U-ExM, we mapped MTOC morphology during mitosis and ciliogenesis (Figure 3D). During mitosis, spindles formed with one centriole in a pair oriented end-on to the spindle (Supplemental Figure S3, C–E). This end-on arrangement has also been observed in other organisms that, like chytrids, have semi-open mitoses with a fenestration of the nuclear envelope at the pole allowing access of microtubules to the nucleoplasm (Powell, 1980; Shah et al., 2024). In late metaphase, as determined by DNA labeling, centriole pairs lacked astral microtubules, but these then appeared in early anaphase as spindle microtubules shorten (Figure 3D). At the end of the mitotic cycles, one centriole elongates to form the basal body (Figure 3D) (Lowry and Roberson, 1997; Lowry et al., 2004) and centrioles had a parallel orientation with bridging centrin signal (Figure 3D; Supplemental Figure S3, C–G). This remodeling of centriole length and orientation occured before changes in the microtubule bundle architecture. Then the centriole at the base of the cilium is surrounded by microtubule bundles that resemble rootlets and terminate at the edge of the centriole (Figure 3, D and E; Supplemental Figure S3A). By the end of ciliogenesis, there is a reduction in the complexity of the microtubule organization until only a single bundle of microtubules emanates from each basal body in the nascent zoospores before their release from the sporangium (Figure 3D).

Chytrid zoospore microtubules appear morphologically similar to microtubule rootlets of other ciliated eukaryotes (Barr, 1981; James et al., 2006; Yubuki and Leander, 2013; Azimzadeh, 2014). The transitions in the chytrid microtubule architecture that we observe during the lifecycle appear similar to that of the slime mold *Physarum polycepharum*, which in its amoeboid lifecycle alternates between a ciliated cell with centriole and rootlet microtubules and a mitotic cell type with a centrosome-like MTOC (Wright et al., 1988). The function of the microtubule bundles in chytrid zoospores and sporangia are not known. The microtubule bundles of the zoospore resemble rootlet microtubules in ciliated microeukaryotes like the green alga *Chlamydomonas reinhardtii* (Mittelmeier et al., 2011; Dutcher and O’Toole, 2016) and may play an analogous role (Galindo et al., 2021) in cell polarity and positioning of organelles or in ciliogenesis to scaffold intracellular or intraflagellar transport. Yet, unlike those of *C. reinhardtii*, chytrid microtubule organization is extensively remodeled to also adopt a geometry resembling a centrosomal aster at some stages of mitosis. In syncytial *Drosophila* embryos, centrosomal microtubule asters ensure proper nuclear spacing and organization of organelles (Lv et al., 2021), although the lack of microtubule asters during metaphase in chytrids suggests that these may not be required at all times to ensure proper spacing. In plants, microtubules often play a key role in cytokinesis, but depolymerization of the microtubule network in the postmitotic sporangia of other zoosporic fungi did not inhibit the analogous process of cellularization (Fisher et al., 2000; Medina et al., 2025).

### Centrioles shorten but are not degraded during ciliary disassembly

The cilium that is assembled at the end of mitosis enables the motility of free-swimming zoospores that are released from the sporangium. However, after chytrid zoospores attach to a substrate or host, they retract their cilium before progressing through their lifecycle (Koch, 1968; Berger et al., 2005; Medina and Buchler, 2020). Previously, we characterized in detail the timeline of chytrid ciliary disassembly and found that over ~2 h cilia are retracted and axonemes are degraded (Venard et al., 2020). Using U-ExM, we were now able to characterize the structure and position of centrioles during ciliary retraction and degradation.

**FIGURE 4:**
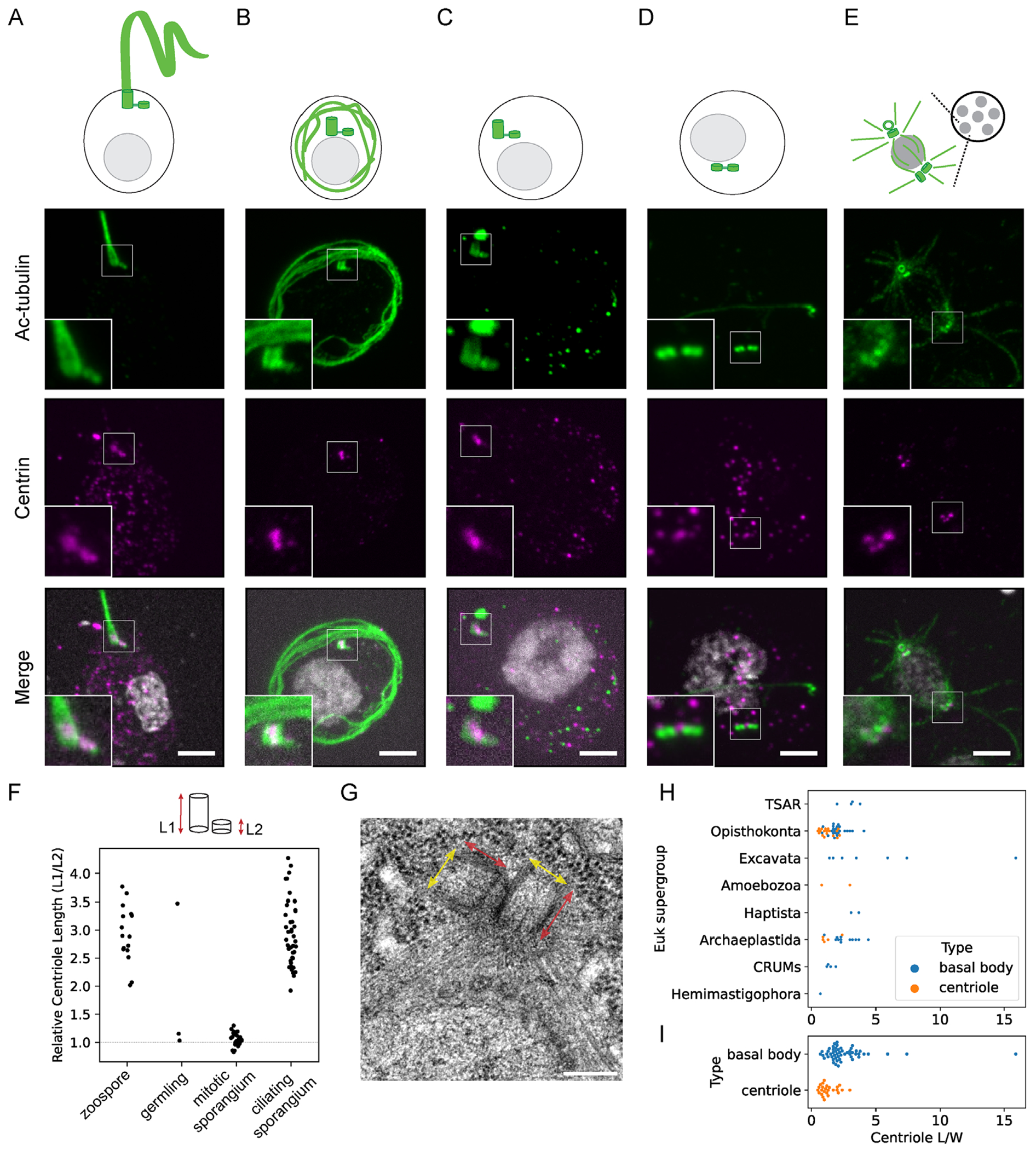
Chytrid fungi detach and shorten centrioles during axoneme degradation. (A–E) Expansion microscopy characterization of chytrid centriole structure during the transition from basal body to mitotic MTOC (acetylated alpha-tubulin; green, centrin; magenta, DAPI/Hoechst; white). Scale bars, 10 μm expanded (expansion factor ~4.5-fold). Insets and summary cartoons show an individual centriole pair from the matched sporangium (insets, 1.5x enlarged). Centrioles are long and parallel in the basal body and then after the centriole pair detaches from the axoneme, the longer centriole shortens in the germling while remaining parallel in orientation, linked by centrin fibers. During the mitotic cell cycles in the sporangium the centrioles are both short, equal in length, and orthogonal to each other. (F) Quantifying the relative centriole length of each centriole pair over the lifecycle shows that in zoospores and the nascent zoospores of the ciliating sporangium, there is asymmetry in centriole length, while in the germling and mitotic sporangia centrioles are short and equal in length. *n* = 16, 3, 26, 41 sporangia, respectively, from three to five independent experiments. (G) TEM image of mitotic *R. globosum* centriole pair showing equal length (red) and width (yellow) centrioles oriented orthogonally; scale bar, 200 nm. (H) Quantification of centriole length in basal body (blue) or centriole (orange) in a range of ciliated eukaryotic species (*n* = 78 centrioles, 51 species). (I) Mean centriole length of basal bodies (blue) versus centrioles (orange) pooled across eukaryotic species in H (*n* = 78 centrioles, 51 species).

In expanded zoospores (Figure 4A), we used an antibody against acetylated alpha-tubulin to label centrioles and observed pairs with one long and one short centriole consistent with the architecture observed by electron microscopy (EM) (Barr and Désaulniers, 1988; Powell et al., 2019) (Supplemental Figure S3A and S3B). Comparing staining with antibodies against acetylated alpha-tubulin versus alpha-tubulin, we find that chytrid centrioles are acetylated all along their length (Supplemental Figure S3C). In most chytrid species, zoospore centrioles have asymmetric length and a parallel orientation (Barr, 1981; Barr and Désaulniers, 1988; Venard et al., 2020). After retraction, we found that the cilium–centriole complex was coiled inside the cell body as the axoneme frayed (Figure 4B). Centrioles separated from the degrading axoneme and remained parallel, apparently linked by centrin-fibers (Figure 4C; Supplemental Figure S2). Instead of being degraded along with the axoneme, centrioles persisted and shortened, forming a pair of centrioles with equal length (Figure 4, C and D). The centriole region that remained was enriched in centrin, consistent with the distribution in the proximal chytrid centriole barrel and with transmission electron micrographs (TEMs) that show proximal structures like the cartwheel (Guichard et al., 2018) are present in both chytrid centrioles in a pair during mitosis (Barr, 1981; Barr and Désaulniers, 1988; Kalbfuss and Gönczy, 2023) (Supplemental Figure S3A). Once the mitotic cycle began, centrioles of the coenocyte were short and orthogonal, and lacked bridging centrin fibers (Figures 4, E and F and 3D; Supplemental Figure S3). By analogy to the centriole cycle of animal cells, this may represent the engaged state (Nigg and Stearns, 2011), which is then followed by disengagement at the end of mitosis.

Reports of centriole shortening as we observed in chytrids are rare (Manton, 1964) as centrioles are normally quite stable structures (Kochanski and Borisy, 1990). In most contexts of cilium disassembly (Liang et al., 2016; Mirvis et al., 2018), cells separate the axoneme and centriole by resorbing the axoneme within an intact ciliary compartment or fully degrade both and synthesize new centrioles *de novo* (e.g., in *Naegleria*) (Fulton and Dingle, 1971; Woglar et al., 2024). Centriole remodeling/reduction is most commonly seen during spermiogenesis (Avidor-Reiss and Fishman, 2019) that involves flaring and loss of the barrel structure, unlike the apparent shortening of the barrel that we observed in chytrid centrioles. The mechanism that might protect a portion of the centriole during degradation of the axoneme in the cytoplasm is not known (Kalbfuss and Gönczy, 2023) but could result from specific posttranslational modifications that protect the centriole or the stabilization of the proximal centriole through the cartwheel or inner centriole scaffold proteins (Le Guennec et al., 2020). Alternately, this remodeling could occur from specific degradation of molecules or structural modules like doublet microtubules that are shared by the axoneme and distal centriole but not the proximal centriole.

### Centriole structure and remodeling in evolution

The barrel structure of the centriole is highly conserved in evolution, yet the centriole remodeling we observed during cilia disassembly in chytrids was striking. We wondered whether this phenomenon of a “short” centriole with roughly equal length and width was an outlier or whether this is revealing some fundamental aspect of centriole biology that is conserved in eukaryotic evolution. We performed a metaanalysis of TEMs of diverse eukaryotic centrioles (Jana, 2021) across all major eukaryotic phyla (Figure 4, G and H; Supplemental Figure S4) and found that the average centriole aspect ratio (length/width) was 2.2 ± 0.2 (*N* = 78, mean ± SEM). The ancestral centriole of the last eukaryotic common ancestor is thought to function exclusively as a basal body (Carvalho-Santos et al., 2011; Mitchell, 2017). We find that centrioles as basal bodies are on average longer than in contexts where they serve as MTOCs separate from the cilium (Figure 4I).

This length difference may be consistent with a loss of constraint on centriole size or a lack of requirement for the distal portion of the centriole when there is no cilium. For example, in the early divisions of the *Drosophila* or *C. elegans* embryo, centrioles remain short, with doublet or singlet MTs, respectively, and a persistent cartwheel structure, and only attain their mature elongated form later in differentiated cell types that will form cilia (Callaini et al., 1997; Pelletier et al., 2006). This is also true during mammalian differentiation, for example, during the development of olfactory sensory neurons, short immature centrioles elongate and gain appendages only in the mature neuron once it is ready to ciliate (Ching et al., 2022). It is possible that the ancestral minimal centriole unit (Azimzadeh, 2021) for transmission through cell division is a small portion of the proximal centriole that lacks distal structures. It is not known whether the centriole is required for the function of the mitotic MTOC in chytrids, but restricting distal centriole assembly to ciliogenesis could serve as a mechanism to ensure separation of MTOC functions between centrosome and basal body at different lifecycle stages.

## CONCLUSION

In this study, we develop the chytrid fungus *Rhizoclosmatium globosum* as a powerful system for studying MTOC remodeling as it undergoes major transitions in centriole and MTOC structure over its synchronous coenocytic lifecycle. Using immunofluorescence, expansion microscopy, and RNA-sequencing we observe substantial remodeling of centrioles and MTOC architecture with different lengths and accessory structures associated with the centrosome-like MTOC versus the basal body as well as dynamic reorganizations of microtubule bundles. The specific remodeling of centriole structure over the lifecycle at the time of ciliogenesis instead of during the cell cycle is quite different from the typical centriole maturation cycle of cultured animal cells. Furthermore, the shortening of the long centriole in the transition from basal body to mitotic MTOC is a surprising and rare form of centriole structural change. Looking forward, new genetic transformation methods in chytrids (Medina et al., 2020; Swafford et al., 2020; Kalinka et al., 2024; Prostak et al., 2023) will allow us to probe the regulatory mechanisms that drive the dramatic remodeling of chytrid centrioles and MTOCs and shed light on fundamental mechanisms for cytoskeletal remodeling and how they emerged in eukaryotic evolution.

## MATERIALS AND METHODS

### Request a protocol through *Bio-protocol*

#### Culture and maintenance of chytrid strains

*Rhizoclosmatium globosum* strain JEL800 (*Rg*) were obtained from J. Longcore (University of Maine, Orono, ME). *Rg* cultures were grown in an incubator at 23°C on solid PmTG medium (0.1% peptonized milk, 0.1% tryptone, 0.5% glucose, and 1% agar). For long-term storage (6 mo), *Rg* cultures were frozen in PmTg medium with 10% DMSO and stored at −80°C or in LN_2_ (Boyle et al., 2003). Every 6 mo, samples were freshly thawed and passaged twice onto solid medium for use in subsequent experiments.

Zoospores were isolated by adding PmTg liquid medium to 2- to 3-d old cultures for 3 to 5 min and then collecting the supernatant. The supernatant was checked under a light microscope for the absence of sporangia, and the concentration of zoospores was measured using a hemocytometer. The zoospores were then concentrated by spinning at 6000 RCF for 15 s. The pelleted zoospores were resuspended, and 2 × 10^5^ spores were plated and incubated at 23°C for 1 to 24 h depending on the experiment.

#### Live cell imaging

Phase-contrast images of the chytrid lifecycle (Figure 1) were acquired with a Keyence microscope with a Nikon 20x/NA objective. Images were acquired for three *z* planes with 1 μm spacing every 5 min for 24 h and the z-plane of best focus was chosen at each timepoint for subsequent analysis.

#### Immunofluorescence

Samples were fixed by the addition of 1.5 ml of 4% formaldehyde directly to the plate. A cell scraper was used to gently dissociate cells from the plate. The fixed cells were transferred into an Eppendorf tube, incubated for 10 min at room temperature, and resuspended in PBS. To remove the cell wall, all timepoints other than 0 min were treated with 50 μg/ml chitinase (Sigma-Aldrich, catalogue no. C6137) in 20 mM potassium phosphate buffer pH 6.0 for 2 h at 45°C. Cells in PBS were then spun-down onto poly-l-lysine–coated coverslips on top of a glass spacer in a leveled round bottom centrifuge tube at 7500 rcf in a Sorvall HB-6 rotor in a Sorval RC-5B centrifuge at 4°C for 20 min. Coverslips were then removed gently from the tubes using a bent paperclip to lift the glass spacers and then postfixed for 20 min in −20°C methanol, and blocked with PBS-BT (1X PBS, 3% BSA, 0.1% Triton-X 100, 0.02% sodium azide) for 1 h. The cells were then stained for 2 h at room temperature with different primary antibodies, and the concentrations used were: acetylated tubulin 6-11B-1 (Sigma-Aldrich, catalogue no. T6793; RRID:AB_609894) 1:1000, *α*-tubulin (Sigma-Aldrich, catalogue no. T6199; RRID:AB_477583) 1:1000, and centrin (EMD Millipore, catalogue no. 04-1624) 1:100. This was followed by incubation with relevant secondary antibodies with 5 μg/ml DAPI for 1 h before mounting into Mowiol on a slide for imaging. The different secondary antibodies (all were in a 1:1000 concentration in PBS-BT) used were: Alexa-Fluor 488 Goat anti-mouse IgG2b antibody (Thermo Fisher Scientific, catalogue no. A-21141; RRID:AB_2535778) and Alexa-Fluor 647 Goat anti-mouse IgG1 antibody (Thermo Fisher Scientific, Cat#A-21240;RRID:AB_2535809).

#### U-ExM

For expansion microscopy, chytrid sporangia were permeabilized through two different freeze-cracking methods rather than with chitinase digestion. In solution, chytrids fixed in 4% PFA in PBS in a microcentrifuge tube for 15 min at room temperature were flash-frozen in a dewar of liquid nitrogen, then thawed in a 37°C or 42°C water bath for 30 to 60 s for between 1 and 10 cycles. Alternatively for freeze-cracking in a thin plane, a ~12-mm circle was drawn with a hydrophobic pen on a glass slide and the center was coated with fresh 1% poly-l-lysine solution for 30 min and rinsed 10 times with water and allowed to air dry for ~10 min. Then 5 to 7 μl of chytrids fixed in 4% PFA in PBS for 15 min at room temperature were added to the center of the slide and sandwiched with a 22 × 50 mm #1.5 coverslip (Thermo Fisher Scientific, 12544B) perpendicular to the slide. This slide sandwich was dipped into liquid nitrogen in a dewar for 10 s or until it stopped bubbling. Then the eraser end of a pencil was used to crack the coverslip off the slide. These slides were postfixed in 100% cold methanol in coplin jars for 10 min then rehydrated in PBS

Fixed and permeabilized cells were incubated either 5 h at 37°C or overnight at 4°C in 0.5 ml freshly prepared acrylamide/formaldehyde solution (AA/FA, 1.4% formaldehyde, 2% acrylamide in PBS) either in a 24-well dish or in a microcentrifuge tube. Gelation was allowed to proceed in monomer solution (19% sodium acrylate, 10% acrylamide, 0.1% bis-acrylamide, 0.5% ammonium persulfate-APS, 0.5% TEMED) on ice for 10 min followed by 1 h at 37°C in a sealed humid chamber and then coverslips were discarded. Gels were boiled at 95°C in 2 ml denaturation buffer (200 mM SDS, 200 mM NaCl, 50 mM Tris pH 9) for 1.5 h. Denaturation buffer was removed, gels were washed with multiple water rinses and allowed to expand in water at room temperature overnight. Small circles (~5 mm in diameter of each expanded gel) were excised and incubated with primary antibodies diluted in PBS-BT buffer (3% BSA, 0.1%Triton X-100 in PBS) on a nutator at 4°C overnight. For experiments in Figures 3 and 4, primary antibodies were acetylated tubulin 6-11B-1 (Sigma-Aldrich, catalogue no. T6793; RRID:AB_609894) 1:1000, *α*-tubulin (Sigma-Aldrich, catalogue no. T6199; RRID:AB_477583) 1:1000, and centrin (EMD Millipore, catalogue no. 04-1624; RRID:AB_10563501) 1:100. The next day, gels were washed three times with PBS-BT buffer and incubated with 5 μg/ml DAPI diluted in PBS-BT and secondary antibodies diluted 1:1000 were Alexa-Fluor 488 Goat anti-mouse IgG2b antibody (Thermo Fisher Scientific, catalogue no. A-21141; RRID:AB_2535778), Alexa-Fluor 488 Goat anti-mouse IgG1 antibody (Thermo Fisher Scientific, catalogue no. A-21121; RRID: AB_2535764), Alexa-Fluor 568 Goat anti-mouse IgG1 antibody (Thermo Fisher Scientific, catalogue no. A-21124; RRID:AB_2535766), Alexa-Fluor 647 Goat anti-mouse IgG2a antibody (Thermo Fisher Scientific, Cat#A-21241; RRID:AB_2535810), Alexa-Fluor 647 Goat anti-mouse IgG2b antibody (Thermo Fisher Scientific, catalogue no. A-21242; AB_2535811). Gels were rocked on a nutator at 4°C overnight. Gels were washed once with 1X PBS and three times with water, and placed in a glass-bottom, freshly poly-l-lysine treated 35-mm dish with #1.5 coverslip bottom for imaging (MatTek corp. P35G-1.5-20-C).

#### Confocal microscopy

For images in Figure 1, slides were imaged using a Leica SP8 scanning confocal microscope with constant exposures during experiments, images were collected using the LAS X program and deconvolved with the HyVolution mode using the in-built Huygens deconvolution software from SVI. For imaging expansion microscopy gels in Figures 3 and 4, samples were imaged with an Zeiss Axio Observer microscope (Carl Zeiss) with a CSU-W1 confocal spinning-disk head (Yokogawa Electric Corporation, Tokyo, Japan), PlanApoChromat 63 ×/1.4 NA oil objective, and a PRIME: BSI backside illuminated CMOS camera run with SlideBook 6 software (3i, Denver, CO). Excitation lasers were 405, 488, 561, and 640 nm. Z-stacks were acquired with 0.4-μm spacing.

#### TEM

For TEM, samples grown for 14 and 16 h after plating were fixed by addition of 1 ml of 2% glutaraldehyde and 4% paraformaldehyde in 0.1 M sodium cacodylate buffer, pH 7.2 on ice for 1 h. After fixation, the cells were spun down 2 min at 6k RPM and resuspended in warm 10% gelatin in PBS for 5 min at 37°C. Samples were postfixed with 1 ml of 1% osmium tetroxide for 1 h on ice. The pellet was washed five times in water, followed by dehydration in an ethanol/water concentration series (50 to 100%). The samples were then washed in a series of propylene oxide/Epon concentrations followed by embedding in Epon. Ultrathin sections were stained with uranyl acetate and lead citrate and analyzed on a JEOL JEM1400 TEM.

#### RNA-sequencing

RNA-sequencing was done for *Rg* grown for 1.5, 13, 17.5, and 22 h after plating zoospores onto solid PmTG medium. We included three biological replicates for each timepoint. For each timepoint, 1 ml of 2 × 10^5^ zoospores/ml were plated on three 15-cm Petri dishes containing 1% PmTG solid medium and incubated at room temperature. Total RNA was extracted at the desired timepoints using TRizol reagent (Thermo Fisher Scientific, catalogue no. 15596026) following the manufacturer’s protocol. This was followed by DNAseI treatment (New England Biolabs, catalogue no. M0303S) and then re-extraction with TRizol. The quality of the total RNA sample was checked using a nanodrop; both A260/280 and A260/230 ratios were above 1.8.

The samples were sent to Novogene Corporation (https://en.novogene.com) for sequencing where they first analyzed RNA quality by Bioanalyzer (Agilent), and then sequenced the samples using the NovaSeq 6000 (Illumina, San Diego, CA), resulting in 150 bp paired-end sequences. Assembly of the sequencing data was performed by Novogene as follows: The raw data were filtered, and the sequences were aligned against the *Rg* reference genome using the program HISAT2. Gene expression levels were analyzed using HTseq software. Differentially expressed genes were identified with DESeq software. Volcano plot, heatmap, Gene Ontology (GO) enrichment, KEGG pathway analysis, and protein–protein interaction network analysis were also performed by Novogene.

For creating Venn diagrams (Supplemental Figure S2B), we filtered for significantly expressed genes above 1 FPKM and input this list to the web tool: http://bioinformatics.psb.ugent.be/webtools/Venn/. Differential analysis of gene expression at each timepoint was done by manually analyzing the highly expressed genes at each timepoint for patterns using GO terms. For the grouped gene categories, ClustVis (Metsalu and Vilo, 2015) was used to generate heatmaps. The FPKM values were ln(x) transformed, rows were centered, and unit variance scaling was applied to rows. For creating the heatmap of the individual genes, no clustering was used (Supplemental Figure S2), whereas rows and columns were clustered using Euclidean distance and average linkage for the averaged values of gene groups (Figure 3, B and C).

#### OrthoFinder analysis

We used a combination of bioinformatic programs to identify the distribution of centriole and centrosome-related protein orthologs across non-dikaryotic fungi. To do so, we downloaded the predicted genomes and proteomes of 213 non-dikaryotic fungi from JGI Mycocosm and 4 outgroups: Animals (*Homo sapiens*), Plants (*Arabidopsis thaliana*), *Naegleria*, and *Dictyostelium*. To guard against false positives, we used a number of quality control steps on the data, resulting in conservative estimates of orthology. First, we removed all datasets with BUSCO scores of less than 80% when compared with the default eukaryotic database (Manni et al., 2021). To generate an initial database of orthologs, we used Orthofinder (Emms and Kelly, 2019) with the remaining high-quality proteomes from JGI (accessed November, 2023). Orthofinder, however, significantly benefits from having a species tree imposed on the input data, but the existing phylogeny of non-dikaryotic fungi is still under frequent revision and does not include many of the species we have in this dataset. To account for this, we took an approach that allowed us to apply constraints from the existing literature and allow the data to inform unresolved relationships. We constructed a backbone phylogeny down to Order using the most currently reviewed phylogeny of non-dikaryotic fungi (Galindo et al., 2023), and then allowed the internal algorithms of Orthofinder to resolve phylogenetic relationships below the level of order.

A limitation and strength of Orthofinder is that it only uses sequence data to determine orthology. Although this makes results easier to interpret, it omits potentially important information that exists for many of these centriole and centrosome-related protein families. The hypothesized orthology of many centriole and centrosome-related proteins in more well-studied organisms leverages additional structural, experimental, and theoretical data. To account for these additional data frequently available in Metazoa and Dikaryotic fungi but conspicuously absent in non-Dikarya, we manually assembled putatively orthologous bait clusters with genes currently hypothesized to be orthologs based on data above the sequence level. Bait protein sequences were downloaded from UNIPROT (https://www.uniprot.org/) as FASTA. Baits include a list of common centriole and centrosome genes (Keller et al., 2005; Carvalho-Santos et al., 2010; 2011; Firat-Karalar et al., 2014; Galindo et al., 2021). Human bait sequences were included as the minimal reference with the exception of zeta-tubulin, where we used the *Xenopus laevis* sequence as there is no copy in humans. To determine broad orthology groupings, we chose to use bait clusters with sequences from multiple organisms that included the two fungi with the best studied MTOCs: *S. pombe* and *S. cerevisiae*. Thus, we included any known orthologs of bait proteins from the yeasts *S. pombe (972 h-)* and *S. cerevisiae (288C)*. Bait clusters also included manually curated orthologs from three zoosporic fungi, *R. globosum (JEL800), B. dendrobatidis (JAM81)*, and *S. punctatus (DAOM BR117)*, where there were significant hits from pHMMER homology prediction and/or reciprocal blastp (NCBI) using the human protein sequence as bait.

We then used these putatively orthologous bait clusters to merge all Orthofinder orthogroups represented by species within the putatively orthologous bait clusters into a single, new merged orthogroup. As expected, many of these putatively orthologous bait clusters mapped to a single Orthofinder orthogroup, but others included two or three Orthofinder orthogroups together. In these cases, we combined Orthofinder orthogroups into a merged orthogroup. Finally, to remove spurious inclusions of protein fragments and off-target inclusions, we imposed a sequence length filter that removed any sequence shorter than 50% of the length of the mean bait sequence length used to create the new merged orthogroup. Each merged orthogroup was then scored against each species in our database, creating a binarized presence/absence database from our conservative estimate of orthologous genes.

#### Data analysis

Lifecycle stages in interphase and mitosis were determined through a combination of sporangium diameter and DNA organization (Figures 1, 3, and 4) as *R. globosum* cell architecture and lifecycle timing is highly stereotyped.

Centriole length and width in expansion microscopy images or TEMs was measured manually with a 1 pixel wide line in a single z plane of best focus using the linescan feature in Fiji version 2.0.0-rc-68/1.52g (Figure 4F; Supplemental Figure S3, B, C, F, and H). Only centrioles where the entire barrel lay along a z-plane were measured to minimize aberrations. For the alpha-tubulin and centrin intensity linescans in Supplemental Figure S3, a five pixel wide line either 2 μm in length or 4 μm in width were drawn across the pair of centrioles in each axis, respectively, and intensity was normalized by background substracting the minimum gray value along the linescan and dividing by the largest difference in gray value along each linescan. Average expansion factor for the dataset was calculated by taking the ratio of the raw centriole width from U-ExM measurements to the raw centriole width from TEM measurements. Microtubule bundle architecture (Figure 3, D and E) was scored manually based on evaluating maximum intensity projections of expanded sporangia to make sure no bundles were excluded. As *Rg* zoospores stereotypically have a single microtubule bundle, we had clear positive examples of this architecture to score late stage sporangia that had cellularized and were preparing to release zoospores. In metaphase (Figure 3D, first column), we scored microtubule bundles as any microtubules that were visible that were not gathered into the spindle pole. This is in contrast with what we observe in anaphase or later stages (Figure 3D, columns 2–4), where microtubules are arranged in aster-like shapes with some radiating toward the chromosomes and others radiating away that we scored as “>1 microtubule bundles” in lieu of scoring an exact number of bundles, which would require a dataset collected in a way that is suited to a quantitative three-dimensional analysis of microtubule intensities. For the centriole angles measured in Supplemental Figure S3D, we drew lines in Fiji down the longitudinal axis of each centriole in a pair and along the pole–pole axis of the spindle and used the “angle” measurement in Fiji to find the angle between each line and horizontal. We then determined the angle between each centriole and the pole–pole axis of the spindle by subtracting the absolute value of these angle measurements, since we did not rotate the images to ensure that each would be in the same quadrant and Fiji reports angles from −180 to 180 degrees. We included four to five spindles per sporangium in five metaphase and once anaphase spindle and only measured linescans in a single z-plane for which the centrioles and both poles of a spindle were all in focus. We scored centriole “1” for each pair as the centriole with the smaller angle relative to the spindle axis.

In the TEM meta-analysis dataset (Figure 4, H and I; Supplemental Figure S4), centriole length is more challenging to score fairly as there is a wide diversity of distal ciliary and transition zone densities. We chose fiducials that avoided the transition zone and were present in as much of the dataset as possible. We measured manually using the linescan feature in Fiji from the proximal to distal end of the centriole and defined the distal endpoint as either the exposed end in a centrosome or the proximal end of any terminal plate structure if present. In the absence of any terminal plate, we measured to the base of the ciliary pocket membrane invagination if present. To maximize the number of images we could include in the dataset, we report that centriole size as an aspect ratio of length divided by width as many TEM images did not contain scale bars or single microtubules of known size to use as a fiducial. We chose at minimum two species per eukaryotic clade across all major kingdoms as well as deep branching eukaryotes of uncertain position (Torruella et al., 2024) (e.g., excavates) in lineages that retain centrioles (Nabais et al., 2020) informed by literature reviews of centriole length across diverse organisms (Hodges et al., 2012; Jana, 2021).

Manual linescan measurements described above were made with Fiji and then imported as CSV files into Python for subsequent analysis. Custom Python scripts written as Jupyter Notebooks (Kluyver et al., 2016) were used to generate all plots.

The raw FASTQ files from the RNA sequencing in this work have been deposited to the NCBI Sequence Read Archive associated with BioProject PRJNA1338479 and are publicly available. All other data and analysis scripts generated in this work are available from the corresponding authors (A.F.L and T.S.) upon request.

## Supplementary Material

Supp

## SIGNIFICANCE STATEMENT

- Cells remodel their cytoskeleton to perform different behaviors. Although we know much about cytoskeletal assembly, we know less about how structures like centrioles are remodeled, for example, to convert between ciliary base and centrosome.
- Using chytrid fungi that retain centrioles and cilia, lost in other fungal lineages, the authors map microtubule organization. They find centrioles are protected during ciliary disassembly, but shorten. Short centrioles form a mitotic centrosome before maturing to reform cilia.
- Centriole remodeling is common in developmental transitions. This work suggests, even in a unicellular organism, coupling remodeling to different lifecycle stages allows cells to dynamically tune cytoskeletal organization.

## Abbreviations used:

MTOC: Microtubule-organizing center
PCM: Pericentriolar material
*Rg*: *Rhizoclosmatium globosum*
U-ExM: Ultrastructure expansion microscopy
