# Supplementary material for: Dynamic remodeling of centrioles and the microtubule cytoskeleton in the lifecycle of chytrid fungi
Source: Mol Biol Cell. Author manuscript; Available in PMC 2026 Mar 3. (PMC12659703; doi:10.1091/mbc.E24-12-0577)
Supplement: Supp [file NIHMS2134680-supplement-Supp.pdf]

Supplemental Materials

Molecular Biology of the Cell

Long *et al*.

Figure S1: Centriole and centrosome gene conservation among chytrid species

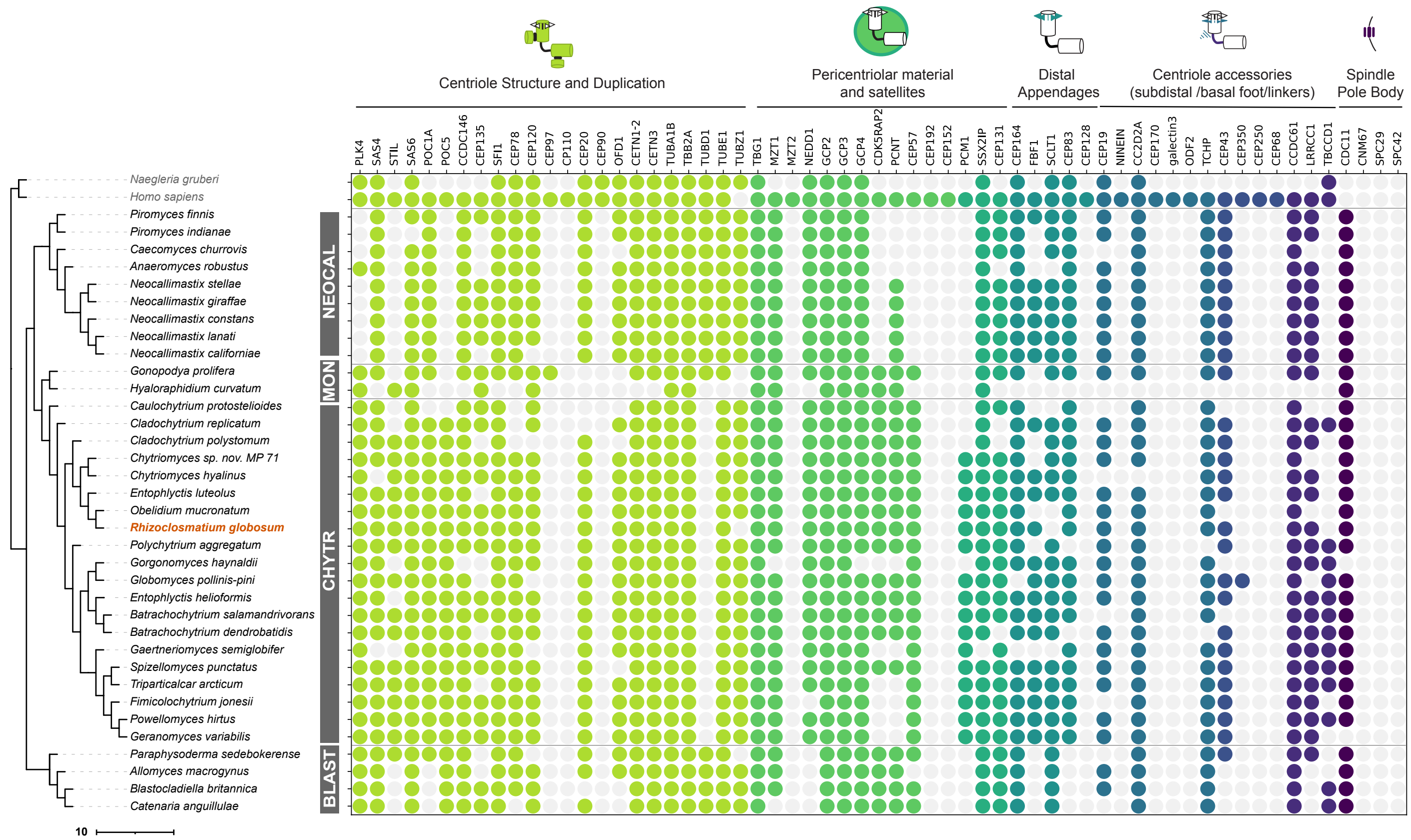

Figure S2: Ordered transcriptional programs underly key cytoskeletal transitions of the chytrid life cycle

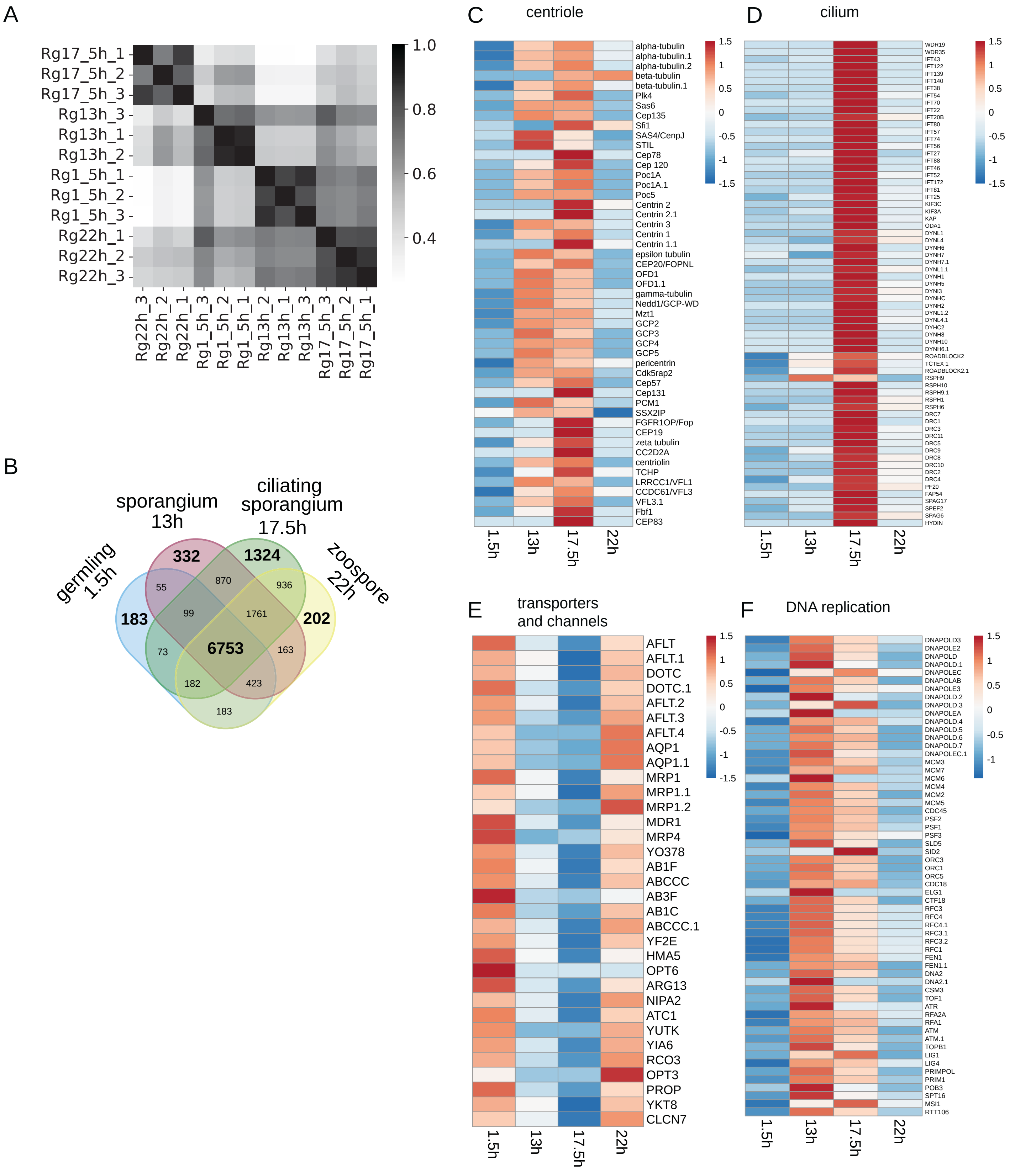

Figure S3: Centriole size, polarity, and orientation change during the chytrid lifecycle

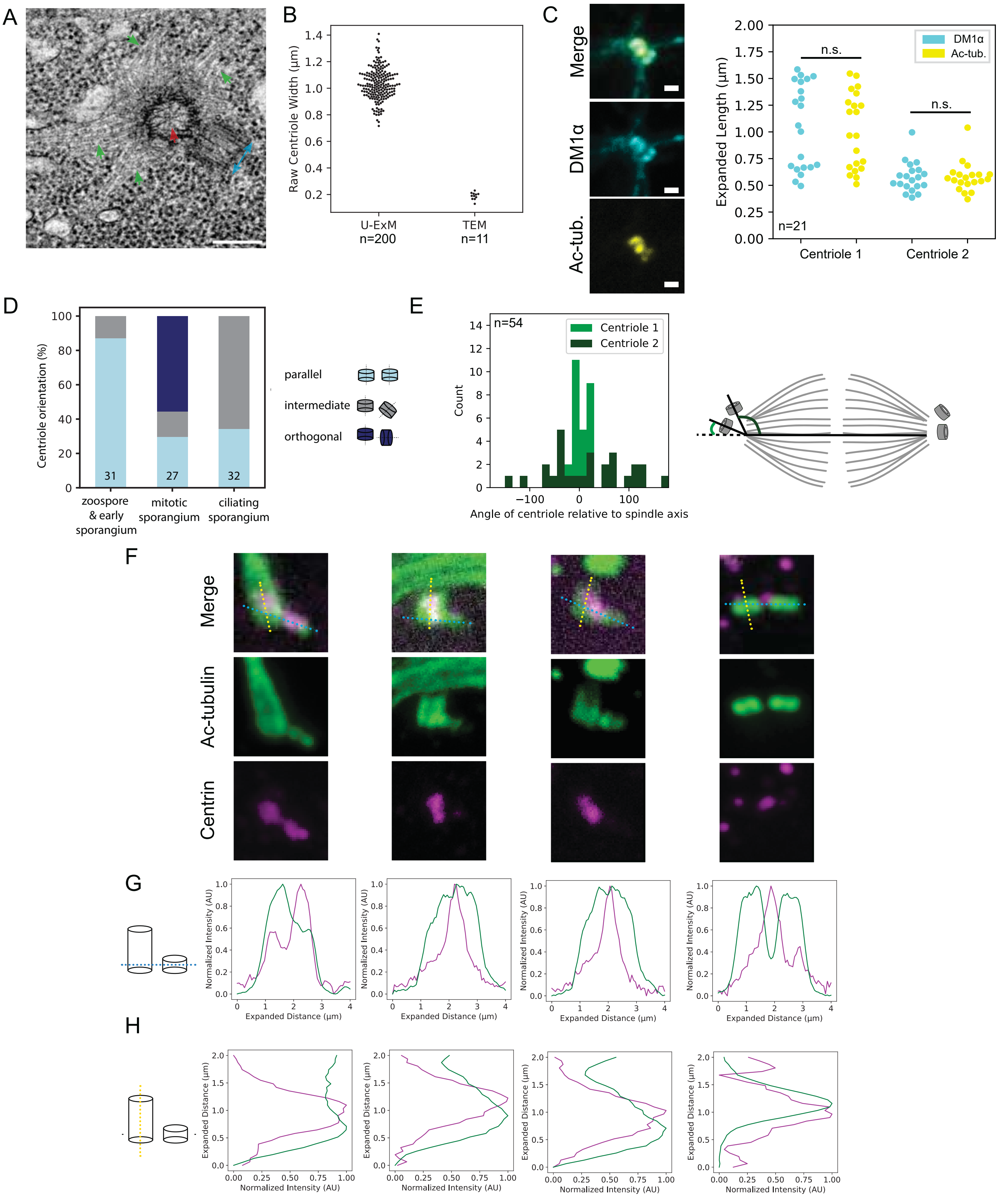

Table S1: Centriole dimensions from electron micrographs.

NCBI ID	Organism	Cell Type	Supergroup	Type	Unit	Centriole length	Centriole width	Centriole L/W	Reference
4785	*Phytophthora cinnamomi*	zoospore	TSAR	basal body	px	128	34	3.8	(Hardham, 1987)
4808	*Blastocladiella emersonii*	sporangium	Opisthokonta	centriole	px	41	57	0.7	(Lessie and Lovett, 1968)
4808	*Blastocladiella emersonii*	sporangium	Opisthokonta	centriole	px	72	60	1.2	(Lessie and Lovett, 1968)
4808	*Blastocladiella emersonii*	zoospore	Opisthokonta	basal body	px	234	79	3.0	(Lessie and Lovett, 1968)
5691	*Trypanosoma brucei*		Excavata	basal body	px	167	68	2.4	(Sherwin and Gull, 1989)
5722	*Trichomonas vaginalis*		Excavata	basal body	nm	616	174	3.5	(Lee *et al.*, 2009)
5741	*Giardia intestinalis*		Excavata	basal body	nm	409	246	1.7	(Nohynková *et al.*, 2006)
5762	*Naegleria gruberi*		Excavata	basal body	nm	281	198	1.4	(Larson and Dingle, 1981)
5791	*Physarum polycephalum*	amoeba	Amoebozoa	centriole	px	148	50	3.0	(Gely and Wright, 1986)
5791	*Physarum polycephalum*	amoeba	Amoebozoa	centriole	px	34	41	0.8	(Gely and Wright, 1986)
5888	*Paramecium tetraurelia*		TSAR	basal body	nm	365	179	2.0	(Bengueddach *et al.*, 2017)
5911	*Tetrahymena thermophila*		TSAR	basal body	nm	434	138	3.1	(Bayless *et al.*, 2015)
13221	*Chrysotila carterae*		Haptista	basal body	px	137	45	3.1	(Beech *et al.*, 1988)
52552	*Mallomonas splendens*		TSAR	basal body	nm	2278	701	3.2	(Beech and Wetherbee, 1990)
55998	*Stigeoclonium sp.*		Archaeplastida	centriole	nm	177	199	0.9	(Manton, 1964)
55998	*Stigeoclonium sp.*		Archaeplastida	basal body	nm	430	202	2.1	(Manton, 1964)
63628	*Trichonympha agilis*		Excavata	basal body	nm	3112	196	15.9	(Nazarov *et al.*, 2020)
63628	*Trichonympha agilis*		Excavata	basal body	px	294	40	7.4	(Grimstone and Gibbons, 1966)
64504	*Allomyces arbusculus*	sporangium	Opisthokonta	centriole	px	60	55	1.1	(Renaud and Swift, 1964)
64504	*Allomyces arbusculus*	sporangium	Opisthokonta	basal body	px	110	53	2.1	(Renaud and Swift, 1964)
65357	*Albugo candida*	sporangium	Opisthokonta	centriole	nm	183	220	0.8	(Berlin and Bowen, 1964)
65357	*Albugo candida*	zoospore	Opisthokonta	basal body	nm	620	240	2.6	(Berlin and Bowen, 1964)
70189	*Olpidium brassicae*	zoospore	Opisthokonta	basal body	px	135	83	1.6	(Temmink and Campbell, 1969)
81100	*Breviata anathema*		Opisthokonta	basal body	nm	436	157	2.8	(Heiss *et al.*, 2013a)
81526	*Monosiga ovata*		Opisthokonta	basal body	nm	376	183	2.1	(Karpov, 2016)
85706	*Ancyromonas sp.*		CRuMs	basal body	nm	269	213	1.3	(Heiss *et al.*, 2011)
104085	*Holomastigotoides sp.*		Excavata	basal body	px	562	95	5.9	(Gibbons and Grimstone, 1960)
109871	*Batrachochytrium dendrobatidis*	zoospore	Opisthokonta	basal body	nm	336	191	1.8	(Longcore *et al.*, 1999)
109876	*Catenaria anguillulae*	sporangium	Opisthokonta	centriole	nm	205	157	1.3	(Ichida and Fuller, 1968)
114254	*Rhizidiomyces apophysatus*	sporangium	Opisthokonta	basal body	px	36	11	3.2	(Fuller and Reichle, 1965)
190325	*Collodictyon triciliatum*		CRuMs	basal body	nm	435	225	1.9	(Brugerolle *et al.*, 2002)
271399	*Sorastrum sp.*	colony	Archaeplastida	centriole	px	61	49	1.3	(Marchant, 1974b)
271399	*Sorastrum sp.*	colony	Archaeplastida	basal body	px	172	89	1.9	(Marchant, 1974b)
287562	*Pediastrum boryanum*	zooid	Archaeplastida	centriole	px	42	44	1.0	(Marchant, 1974a)
287562	*Pediastrum boryanum*	zooid	Archaeplastida	basal body	px	87	39	2.3	(Marchant, 1974a)
301696	*Monoblepharis polymorpha*	zoospore	Opisthokonta	basal body	nm	379	218	1.7	(Mollicone and Longcore, 1994)
329046	*Rhizoclosmatium globosum*	zoospore	Opisthokonta	basal body	px	93	55	1.7	(Powell *et al.*, 2019)
329046	*Rhizoclosmatium globosum*		Opisthokonta	centriole	px	47	46	1.0	This work.
424538	*Cricosphaera elongata*		Haptista	basal body	nm	1482	399	3.7	(Henry *et al.*, 1991)
529818	*Thecamonas trahens*		Opisthokonta	basal body	nm	339	265	1.3	(Heiss *et al.*, 2013b)
906914	*Chlamydomonas reinhardtii*		Archaeplastida	basal body	nm	358	183	2.0	(Dutcher and O'Toole, 2016)
2012329	*Olpidium cucurbitacearum*	zoospore	Opisthokonta	basal body	nm	328	177	1.9	(Barr and Hadland-Hartmann, 1977)

NCBI ID	Organism	Cell Type	Supergroup	Type	Unit	Centriole length	Centriole width	Centriole L/W	Reference
2012329	*Olpidium cucurbitacearum*	zoospore	Opisthokonta	centriole	nm	119	217	0.5	(Barr and Hadland-Hartmann, 1977)
2027450	*Hemimastix amphikineta*		Hemimastigophora	basal body	nm	135	190	0.7	(Foissner *et al.*, 1988)
2823256	*Caraotamonas croatica*		CRuMs	basal body	nm	184	126	1.5	(Yubuki *et al.*, Nov-Dec 2023)
2823262	*Fabomonas mesopelagica*		CRuMs	basal body	nm	149	127	1.2	(Yubuki *et al.*, Nov-Dec 2023)
173374	*Trentepohlia aurea*		Archaeplastida	basal body	px	131	57	2.3	(Graham and McBride, 1975)
13778	*Nitella sp.*		Archaeplastida	centriole	px	53	59	0.9	(Turner, 1968)
13778	*Nitella sp.*		Archaeplastida	basal body	px	152	47	3.2	(Turner, 1968)
3196	*Marchantia sp.*		Archaeplastida	basal body	px	128	37	3.46	(Carothers and Kreitner, 1968)
67246	*Aulacomnium palustre*		Archaeplastida	centriole	nm	448	190	2.4	(Bernhard and Renzaglia, 1995)
13804	*Sphagnum sp.*		Archaeplastida	basal body	px	27.2	27.9	1.0	(Manton, 1957)
87755	*Phaeoceros sp.*		Archaeplastida	basal body	px	50	21.6	2.3	(Carothers *et al.*, 1977)
3251	*Lycopodium sp.*		Archaeplastida	basal body	px	111	25	4.44	(Carothers *et al.*, 1975)
3257	*Equisetum sp.*		Archaeplastida	basal body	px	99	33	3	(Duckett and Bell, 1977)
3239	*Psilotum sp.*		Archaeplastida	basal body	nm	684	186	3.7	(Renzaglia *et al.*, 2001)
9606	*Homo sapiens*	breast epithelial	Opisthokonta	centriole	nm	397	190	2.1	(Lingle *et al.*, 1998)
9606	*Homo sapiens*	U2OS	Opisthokonta	centriole	nm	382	189	2.02	(Arquint *et al.*, 2012)
9606	*Homo sapiens*	RPE-1	Opisthokonta	centriole	nm	434	201	2.2	(Marteil *et al.*, 2018)
9606	*Homo sapiens*	RPE-1	Opisthokonta	basal body	nm	342	158	2.2	(Molla-Herman *et al.*, 2010)
10090	*Mus musculus*	olfactory epithelial	Opisthokonta	centriole	nm	190	190	1	(Falk *et al.*, 2015)
10090	*Mus musculus*	olfactory epithelial	Opisthokonta	basal body	nm	412	190	2.2	(Falk *et al.*, 2015)
10090	*Mus musculus*	photoreceptor	Opisthokonta	basal body	nm	410	209	2.0	(Falk *et al.*, 2015)
10090	*Mus musculus*	trachea	Opisthokonta	basal body	nm	423	235	1.8	(Burke *et al.*, 2014)
7955	*Danio rerio*	larval brain	Opisthokonta	basal body	nm	259	154	1.7	(Wilkinson *et al.*, 2009)
7955	*Danio rerio*	neuroepithelium	Opisthokonta	centriole	nm	273	163	1.7	(Dzafic *et al.*, 2015)
9031	*Gallus domesticus*	choroid plexus	Opisthokonta	basal body	nm	1506	793	1.9	(Stephen *et al.*, 2015)
9031	*Gallus domesticus*	choroid plexus	Opisthokonta	centriole	nm	537	274	2.0	(Stephen *et al.*, 2015)
6239	*Caenorhabditis elegans*	embryo	Opisthokonta	centriole	nm	208	177	1.2	(Pelletier *et al.*, 2006)
7227	*Drosophila melanogaster*	auditory neurons	Opisthokonta	basal body	nm	196	86	2	(Jana *et al.*, 2018)
7227	*Drosophila melanogaster*	spermatocyte	Opisthokonta	basal body	nm	1667	410	4.1	(Jana *et al.*, 2018)
7227	*Drosophila melanogaster*	larval wing disc	Opisthokonta	centriole	nm	124	143	0.87	(Franz *et al.*, 2013)
7227	*Drosophila melanogaster*	larval brain	Opisthokonta	centriole	nm	93	157	0.59	(Franz *et al.*, 2013)
7227	*Drosophila melanogaster*	olfactory neuron	Opisthokonta	basal body	nm	173	208	0.83	(Gottardo *et al.*, 2015)
7227	*Drosophila melanogaster*	gonioblast	Opisthokonta	basal body	nm	168	187	0.9	(Gottardo *et al.*, 2015)
7227	*Drosophila melanogaster*	spermatogone	Opisthokonta	basal body	nm	341	198	1.72	(Gottardo *et al.*, 2015)
76800	*Anisopteromalus calandrae*	larval cell	Opisthokonta	centriole	nm	137	217	0.6	(Uzbekov *et al.*, 2018)
76800	*Anisopteromalus calandrae*	spermatid	Opisthokonta	centriole	nm	275	197	1.40	(Uzbekov *et al.*, 2018)

Figure S1: Centriole and centrosome gene conservation among chytrid species
Individual species level OrthoFinder analysis, supporting the phylum level trends in Figure 2, showing orthologs of genes associated with different centriole and centrosome features across two outgroups (top, gray) that have centrioles and motile cilia and all of the individual species included in our dataset within the zoosporic clades of fungi (NEOCAL, Neocallimastigomycetes; MON, Monoblepharidomycetes; CHYTR, Chytridiomycetes; BLAST, Blastocladiomycota). Each colored circle denotes that there was at least one ortholog assignment in the species. *R. globosum*, the organism in this study, is highlighted in brown.

Figure S2: Ordered transcriptional programs underly key cytoskeletal transitions of the chytrid life cycle. A) RNA sequencing of three biological replicates were performed for each of 4 timepoints. Heatmaps of the Pearson correlation coefficients for each sample show that biological replicates are highly correlated (scale 0, white to 1, black). B) Venn diagram showing unique and shared transcripts between four different timepoints in the lifecycle (1.5h, 13h, 17.5h, 22h). C-F) Heatmaps of individual genes showing transcript levels at four timepoints in the lifecycle. The groupings in C and D correspond to the individual genes averaged in Figure 3B,C. Groupings in E and F confirm that expected transcripts associated with DNA replication and channels and transporters are upregulated in timepoints with maturing sporangia and zoospore/germling respectively.

Figure S3: Centriole size, polarity, and orientation during the chytrid life cycle.
A) TEM of orthogonal pair of centrioles from *R. globosum* (200 nm scale) showing presence of cartwheel (red arrow) in short centrioles (blue arrow denotes centriole width). Multiple microtubule bundles (green arrow) surround the centriole. B) Centriole width in micrometers measured from U-ExM gels (n=200 centrioles) versus TEM (n=11 centrioles) showing average expansion factor of 5.5 ± 0.2 (mean ± std). C) Quantification of centriole length measured with alpha-tubulin (cyan) versus aceylated alpha-tubulin (yellow) labels supports that centriole length measurement is not significantly affected by labeling strategy (n=21 centriole pairs from 21 sporangia, Centriole 1 DM1α vs Ac. Tub, W = 74, p=0.16; Centriole 2 DM1α vs Ac. Tub, W = 91.5, p=0.89, Wilcoxon-signed rank test). Scale bar 1 μm. D) Quantification of centriole pair orientation over the chytrid lifecycle (light blue: parallel, dark blue: orthogonal, gray: intermediate) showing that orthogonally oriented centrioles are only found in mitotic sporangia. E) Quantification of longitudinal centriole angles (degrees) relative to the pole-pole axis of the

spindle in metaphase and anaphase sporangia (n = 54 spindles from 6 sporangia). F) Immunofluorescence images of expanded chytrid centriole pairs (matching insets in Figure 4A-D) showing alpha-tubulin (green) and centrin (magenta). G,H) Linescans of fluorescence intensity corresponding to images in (F) along width (blue) and length (yellow) of centriole pairs as diagrammed in each cartoon.

Table S1: Centriole dimensions from electron micrographs.
